# Dr. Barlow on the Physiology and Pathology of the Animal Frame

**Published:** 1823-09-01

**Authors:** 


*An Essay on the Medicinal Efficacy and Employment of the Bath Waters; illustrated by Remarks on the Physiology and Pathology of the Animal Frame, with reference to the Treatment of Gout, Rheumatism, Palsy, and Eruptive Diseases.* By EDWARD BARLOW, M.D. Graduate of the University of Edinburgh; Member of the Royal College of Surgeons of Ireland; one of the Physicians of the Bath Hospital, and of the Bath City Infirmary and Dispensary; and Physician of the Charitable Society for the Relief of Lying-in Women. Octavo, pp. 200, Bath, 1822.

DR. BARLOW justly remarks that a Treatise on Mineral Waters, by a Physician residing at their Source, is too apt to be regarded either as an attempt to bring the author into notice, or so to exaggerate the medicinal virtues of the springs as to entice visitors to the spot. At the same time it is highly probable, that the fear of this imputation has prevented many able but modest physicians giving publicity to valuable information acquired by local experience. To omissions of this kind, our author thinks, may be attributed much of that decline, in public and professional estimation, which the Bath Waters have experienced of late years—a decline, we imagine, not a little accelerated by the undissembled scepticism of some eminent physicians on the spot, especially the late Dr. Parry. The object of Dr. Barlow's present work is not to go over the long beaten track of chemical analysis, and dissertations on the well-known general properties of the Bath waters; but rather to shew that their administration in several diseases is consistent with the best established principles of pathology, and not incompatible, even with those peculiar doctrines which have been considered as chiefly influential in bringing them into disuse. He hopes, also, by appeals to actual experience, to prove the advantage of these waters in cases where the presence of inflammatory symptoms would appear to contra-indicate their use.

But, as mineral waters, like all other medicinal substances, derive their efficacy from the powers which they possess of calling into action the inherent energies of the living system, so it is necessary to study the physiology and pathology of the animal frame in connexion with any investigation of the said waters, and this is what Dr. Barlow has done. In fact, if all that is directly applicable to the mineral springs of Bath was expunged from the volume before us, there would be no great deficit in its size, or perhaps in its real value. We may, indeed, observe here that a more extended view of our author's physiological and pathological principles was published several years ago in the 9th and 10th volumes of the Edinburgh Medical and Surgical Journal. His subsequent experience, he observes, founded on a tolerably extensive public and private practice, has confirmed his convictions of the soundness of his published principles, pathological and practical.

As these principles appear to us to contain much that is valuable, though perhaps not much that is entirely new, we shall not deem it inexpedient to give a rapid coup d'œil of them in this place.

1. *General Pathology*. The most prominent, and not the least surprising feature in the animal frame, is the constant and simultaneous processes of renovation and decay which go on from birth till death. The states of assimilation and secretion vary very much, however, and are, in civilized life at least, very seldom in the just and salutary equilibrium necessary for the support of health. If the balance is in favour of assimilation, repletion must necessarily succeed; but this last may, and often does take place under a moderate and even abstemious supply of food, when, from a sedentary life, inactive habits, or defect in some of the emunctories, the necessary quantum of excretion or secretion does not obtain.

"As repletion, then, may take place under very different circumstances, so is its presence marked by very different phenomena. Whenever it arises one of two consequences is sure to result: either it excites the several functions, if sufficiently healthy and vigorous, to increased actions tending to its speedy appropriation and removal, or if these functions be weakly and unable at the moment to institute and support those depuratory processes by which the animal frame is enabled to throw off noxious or redundant matter, then the functions of life, oppressed by a task to which they are unequal, manifest a decline of ordinary power, and all the outward phenomena of debility are displayed. To discriminate this state from one of real debility, as arising from exhaustion of animal power, or defect of nutriment, is a matter of practical importance, not inferior to any which medical science may be engaged in illustrating." 13.

This is an extremely important subject, and our time will not be uselessly employed in taking a somewhat nearer view of it in this place.

When redundancy of nutrition obtains in a healthy constitution, its primary effects are manifested in what may be termed an exuberance of health, rather than a state of disease. The functions are vigorously performed—the nutrition of the several structures is above par—and an increase of bulk is the result, especially if the habits of the individual are not unusually active. If the excess be casual, or inconsiderable in extent, the self-adjusting powers of the constitution may dispose of it so as not to end in actual disease. But if the extent or continuance of this excess urge the powers beyond a certain point, the sanative efforts of the constitution fail, and irregular actions of various kinds obtain, laying the foundation of a large proportion of the specific diseases to which human nature is liable. The general character of diseases so occurring is inflammatory, and the specific form and seat will depend on the predisposition of the system, the accidental debility of particular organs, and the casual excitement to which the body may be subjected. It is needless to remark that depletion, positive and negative, is the remedy for this state of things.

The interval which precedes the occurrence of these increased actions in weakly habits, where the self-adjusting powers are unequal to early restoration, is one of peculiar interest, because its phenomena are of equivocal character, resembling in many respects those which belong to a very different state of the system, and, hence, liable to be misunderstood. They have hitherto been very imperfectly investigated or described. In fact, the attendance of the physician is seldom required until this incipient derangement has passed into some more distinct form of disease. On this account our author has entered more fully into its consideration, pathological and therapeutical, than would be necessary in discussing other and more familiar forms of disease. It is scarcely less important to study with care also that state of the system in which repletion takes place under moderate nutrition with defective secretion, and in which the system is oppressed, not by the quantity of nutriment or the labour of disposing of it, but by the load of excrementitious matter undischarged. Our author now proceeds to consider the three several conditions of plethora which have just been noticed.

To the first condition, or that arising from excess of nutrition in a healthy subject belong all the cases of pure inflammation which we daily witness—the specific form of the disease arising partly from the operation of the exciting cause, but chiefly from the predisposition of the parts affected.

"When, from the increase or continuance of a state of plethôra, the powers of the constitution fail to dispose of the excessive nutriment by applying it to a more abundant nutrition of the several structures of the body, the increased efforts subside for a time, and a state of incipient debility ensues: the pulse becomes low, oppressed, irregular. If this state be relieved by adequate evacuations and a reduced diet, the plethora is removed, and health is restored; no specific disease ensuing. But, if neglected, it either passes, sooner or later, into a state of permanently increased action, amounting to fever or inflammation, and some specific disease is induced, according as the constitution is predisposed to any particular malady; or else, if the constituation be, from natural delicacy, or the extent of labour to be performed, unequal to the effort necessary to generate a state of fever or inflammation, the general health progressively declines, the powers of life become enfeebled, and the constitution sinks under some of the complicated forms of chronic disease." 23.

The acute attack itself is easily recognized and soon subjected to proper medical treatment; yet, previously to this, a deviation from a state of health may generally be detected, which as clearly indicates the propriety of some depletion, though perhaps to a moderate extent, as when the acute attack has actually supervened. If this state were relieved by adequate depletion and other suitable means, the said acute attack would be always prevented, or so mitigated as to be free from danger. We need hardly observe that as these states quickly terminate in death or recovery, they rarely admit of the use of the Bath-waters. We therefore proceed to consider the second state of plethora, or that in which repletion takes place in a constitution deficient in natural vigour, or impaired by predisposition to disease.

"In this state, the earlier deviations from a healthy condition take place so gradually, that they are for a long time nearly unnoticed: there is occasional languor and disinclination to the customary exertions; irregular distribution of blood as marked by coldness of feet and variable countenance, the bowels are irregular, the appetite is capricious, or fails; and both flesh and strength decline. At the earlier periods of this state, the pulse will be found weak, often irregular. Sooner or later a febrile or inflammatory state ensues, marked by a quickened circulation, some increase of temperature, and a white or furred tongue. This state may continue for an indefinite period, and be subject to frequent variations. Most frequently in course of time some local ailment supervenes, which engrosses the attention, and to which, when discovered, the constitutional indisposition is not unfrequently ascribed." 25.

In order to form a clear conception of the phenomena characterising this state of plethora, great attention must be paid to the pulse. Its incipient lowness is that symptom which so generally misleads, as it suggests the employment of tonic and stimulant medicines. It is highly necessary, therefore, to distinguish it from a pulse of pure debility; and this, our author thinks, may be done by conducting the examination with suitable accuracy.

"'If firm pressure be made on the artery, it will be found to resist this, and, on gradually withdrawing the pressure, to rebound against the finger with a force much more considerable than a casual examination would lead one to expect.' With respect to the irregularity of the pulse, I also noticed that this could be observed as occurring both in its force and frequency, and that the irregularity of force, or that in which the artery makes a few strong pulsations, as if by a sudden effort, and again relapses into a state of diminished and oppressed action, indicates a nearer approach to the period of actual fever or inflammation than the regularity of frenquency only. Indeed it seems to be the connecting link between the stages of feebleness and of permanently increased action, and to consist essentially in the desultory and imperfect efforts of the vascular system to form this latter stage; for when the febrile or inflammatory stage is fully formed, the irregularity, for the most part, disappears" 27.

Therapeutical observations will be made farther on. We must therefore pass to the consideration of the third or last stage of plethora, in which the repletion arises, not from excessive nutrition, but from defective excretion, and consequent accumulation of matters that ought to be expelled the system.

This deviation from health takes place still more gradually than in the former instances. As exercise is the principal stimulant for exciting the various secretions, this state of repletion exists more commonly among the class of sedentary and indolent individuals.

"It is characterised by great sallowness of aspect, and duskiness of skin; the pulse is low and compressible; the surface is in general harsh, dry, and deficient in natural transpiration; the tongue, for the most part, moist and clean; the appetite capricious, often craving and voracious; the alvine evacuations perseveringly foul, and exhibiting no traces of healthy secretion; the urine high coloured, and often excessively fetid: even the perspiration has frequently an offensive odour, and gives a dusky tinge to the linen which absorbs it." 29.

Our author knows of no symptom which so strikingly characterises this state as the obstinate foulness of the bowels which no employment of purgatives seems capable of correcting.

"If the use of purgatives happily give such relief to the system as to enable it to make an effort towards forming a febrile or inflammatory stage, if the pulse become quicker and firmer, the tongue white and loaded, with such evidences of increased activity of circulation as to warrant the use of small blood-lettings, these, if employed with sound discretion, and adapted to the powers of the constitution, will do more to correct the morbid condition of the bowels, and bring back healthy evacuations, than any use of purgatives, however judiciously managed, or perseveringly employed." 29.

This fact Dr. Barlow has so very frequently witnessed that, whenever the bowels are found to resist the purgative treatment, he can confidently recommend a moderate use of the lancet as the most effectual auxiliary that can be employed. This measure, however, requires caution; for if resorted to at a very early period, while the powers of life are still torpid and oppressed, or if pushed beyond the normal line, much mischief may be done, and irremediable exhaustion may ensue.

We must pass over Dr. Barlow's remarks on the treatment of inflammatory affections as they occur in the first condition of plethora before described. They are very judicious, and deserve the undivided attention of the rising generation of the profession. The practice he inculcates is bold and decisive, but it is in circumstances which bear and demand the most vigorous depletion.

We now come to the medical treatment of the second condition of plethora—that in which repletion takes place in a constitution deficient in natural vigour, or impaired by previous disease.

As this occurs oftener than the former, and has a greater number of diseases connected with it, it is of more importance, and requires closer attention. In this, as in the first condition of plethora, the earliest deviation from health is marked by declining power in the general circulation. When this stage of feebleness is prolonged for any time, the defective excretory functions often give rise to more or less excrementitious accumulation, and thus a combination of the second and third conditions results.

"More frequently, however, this feebleness is superseded by increased action, and some congestion, inflammation, or other local malady, becomes superadded. A hard and frequent pulse, increased heat of skin, and whiteness of tongue, mark the formation of the second stage; and if unexasperated by stimulant regimen, this may, in a constitution not predisposed to any particular malady, continue for months, or even years, without specific disease ensuing. I have so repeatedly witnessed this, that I have long been in the habit of distinguishing this state by the term "constitutional inflammation;" meaning thereby to designate general inflammatory action in the system, unattended by any local inflammation.

"All the phenomena which belong to this stage are of an inflammatory character: blood drawn frequently exhibits a thick buffy coat, cupped, and with contracted edges; and the treatment of inflammation is that only which affords effectual relief. If taken at an early period, moderate depletion, and antiphlogistic regimen, will speedily relieve it; but if neglected, or improperly treated by tonics and stimulants for any length of time, it becomes more difficult to remove, acquiring obstinacy from its continuance, and laying the foundation of various specific diseases of the worst kind. The treatment of the several stages will require to be discussed at some length, and copiously illustrated." 49.

That nutritive plethora may, in its simplest form and earliest stage, be relieved by artificial means, without any excitement of the system, our author has unequivocally experienced in his own person; and has thus been able to satisfy himself of a fact which there are few opportunities of witnessing. Here he alludes to the popular belief in the utility of of periodical blood-letting in spring and fall, even where there is no disease present in the system, and seems not inclined to question the validity of the belief, or, as it is generally termed, prejudice. "In the luxurious habits of modern times, so condusive to plethora, a discriminating use of this practice might perhaps be beneficially revived."

Under the second condition of plethora he conceives that the congestions and other derangements of minute structure and functions are not to be removed without some effort of the constitution, and therefore that febrile excitement is a valuable auxiliary in effecting recovery. Hence we may often excite and deplete at the same time, without involving any contradiction of principle.

"I am certain, from my own observation, that when the pulse becomes irregular, blood may be taken, not only with safety, but advantage; and that the relief afforded will be speedily manifested by the increased frequency and tone which the pulse acquires. For this purpose the earlier blood-lettings must be small: from four to six ounces is the quantity I usually direct. Its repetition will, of course, depend on the effects; and there can be no more interesting subject in pathology than to mark in the extreme cases of such condition how progressively, under repeated venesection, the pulse rises, and the buffy coat is displayed on the blood. Some striking instances of this kind I have recorded in my former essay. In one, an enfeebled and emaciated boy, labouring under diabetes, lost 209 ozs. of blood in twelve successive blood-lettings; the blood becoming changed from a dark grumous coagulum of loose texture, to the thickest and firmest buff; and the strength of the body increased from feebleness, hardly admitting of an erect posture, to a degree of vigour enabling him to hold the plough for several hours a day. In another, a weakly and delicate female, without any local disease, save alternating pains of head and chest, 106 ozs. were taken in seven blood-lettings, with similar changes in the blood, and as well marked improvement of general strength. This person is alive at the present day, an example of the efficacy of her former discipline; and, excepting a local malady, wholly unconnected with her former disease, is in robust health. Such instances must be deemed conclusive of the soundness of the pathology which I inculcate. Their early symptoms were those of seeming debility; yet if this had been real, life must inevitably have been destroyed by the means employed to preserve it. If, then, there are states of disease marked by considerable debility, in which the constitution not only bears depletion without sinking, but, on the contrary, acquires very considerable accession of strength from copious evacuations, it surely is of the first importance to medical science that such diseases should be scrutinized, and more adequately illustrated than they have hitherto been." 52.

When, by the judicious combination of small bleedings and moderate excitement, the system is aroused to a state of inflammatory action, the treatment will then be obvious enough to any judicious practitioner.

"In judging of its approach, I am influenced more by the state of the tongue, than that of the pulse. When the constitution is assuming a disposition to febrile excitement, ere it is announced by the pulse still feeble and compressible, the tongue presents a peculiar whiteness, strongly characteristic and expressive, being distinct from any obvious morbid secretion, and seemingly belonging to the substance of the tongue itself. It seems in proof of this whiteness not arising from morbid secretion, that I have repeatedly seen it disappear immediately after a full blood-letting. I know not any stronger or surer evidence of inflammatory action than this appearance of the tongue; and I consider that whenever it is present, blood-letting may be had recourse to with little hesitation." 53.

As in this state of plethora a certain degree of excitement is required in aid of early depletion, our author knows of no remedy so suitable for this purpose as the Bath waters. It is in the various modifications of this constitutional derangement that these waters are so eminently beneficial, combined with suitable evacuations, when low inflammatory action is present.

"When constitutional inflammation is present, and still more when local inflammation is superadded, I have for years been in the habit of bleeding, according to the exigences of each case, without finding it requisite to suspend the use of the waters beyond the day on which the blood is taken. In aid of such depletion the medicine usually administered is, a solution of Epsom salt and tartar emetic, given, in divided doses, as an ordinary saline. By the united employment of this remedy and blood-letting, are patients enabled to continue the use of the waters with safety and advantage under every stage of constitutional inflammation, as marked by a quickened circulation, increased heat of skin, and white tongue, who, if depletions were not conjoined, would be obliged to relinquish the use of the waters for long intervals, and thus be deprived of much of the benefit which the diseases admitted into this hospital so signally experience." 55.

Thus then it appears that blood-letting is a remedy suited to each stage of this condition of plethora. In the stage of feebleness, when long protracted, small evacuations of blood relieve the oppressed constitution, and enable it to bring out a salutary degree of excitement, so necessary for correcting the derangements of the system.

"When used for this purpose, the early depletions should never exceed a few ounces, from four to six being a full blood-letting. According as the stage of excitement advances, more copious depletion will be not only borne but required. The object of the incipient bleedings is, not to make that impression on the moving powers which is necessary for the abatement of inflammatory action, but merely to abstract a portion of the circulating fluid, and thus lessen the plethora by which the system is oppressed. The blood may, therefore, be taken from a small orifice, and in a recumbent posture. According as the period of excitement approaches, the effect of depletion in hastening its advancement becomes more manifest; and indeed it is a matter of familiar observation to surgeons, that even while the blood yet flows from the vein, the arterial powers so sensibly increase, that the tardy and sluggish stream, which at first only trickled down the arm, becomes converted to a full and vigorous jet. by which the blood is oftentimes propelled to a distance of several feet. When this degree of increased action is displayed, blood may be more freely taken, and antiphlogistic regimen more rigorously pursued." 56.

During the second stage it is clear that the object of venesection is both to abstract blood and moderate inflammatory action. Wholly or suddenly to subdue this action is not intended; and therefore moderate blood-lettings are most suitable. Eight or ten ounces, Dr. Barlow observes, will often suffice, and from twelve to sixteen will very frequently be well borne.

Our author makes many judicious remarks on purging—the evacuation next in power to blood-letting. The term is very vaguely employed, and comprehends processes essentially different. The attentive practitioner knows that some medicines only stimulate the intestines to dislodge their contents—that some cause the mucous follicles to secrete a considerable quantity of fluids—and that other medicines have the power of drawing into action the glandular organs of the abdomen, as the liver, pancreas, &c. and thus of pouring forth various secretions into the intestinal canal to be evacuated per anum. These processes are, of course, very different, not only in degree, but in kind, and ought to be discriminated when we talk of "purging."

"Consistently with these views, it would appear that the most perfect evacuation of the whole canal must be that procured by combining medicines of each class; and we accordingly find the purgatives in most general use so constituted. When full purging is required, to allay fever, or lessen arterial action, no practice is more common than to give at night a suitable dose of the extract of aloes or colocynth, which are simple aperients, combined with calomel, or calomel and antimony, medicines that produce mucous secretions, and followed next morning with a saline purgative, which, while it accelerates the operation of the previous dose, produces also copious watery stools. When these are not required, but yet a state of disordered intestinal secretion is manifested, it is often expedient to correct this latter gradually, the nature and activity of the cathartic employed being suited to the particular design; and whether it consist of colocynth, calomel and antimony, or rhubarb, blue pill, and ipecacuan, the same principles regulate its administration, and similar effects are produced, differing in degree only.

"With these principles to guide us in the use of purgative remedies, they may be so employed as to be most powerful auxiliaries of blood-letting, for relieving the second condition of plethora in its several stages. In the earlier stage, simple aperients, combined with mercurial and antimonial preparations, in moderate doses, are the most suitable. Accordingly as the febrile stage succeeds, saline purges may be more freely employed; and when local inflammations arise, these latter are indispensable." 61.

These preliminary remarks, and other judicious observations which our author has made on purgatives and sanguineous depletion, clear the way for his investigation of the nature and treatment of some specific forms of disease to which the Bath Waters have been generally found applicable. Of these, Gout naturally comes in for the first share of attention.

### GOUT.

All men know that this disease is characterized by a state of vague indisposition, succeeded by local inflammation of a peculiar kind, on the exposition of which, the previous derangements become sensibly relieved, and on the subsidence of which, the general health becomes restored, with the exception of some degree of debility in the affected joints. It is too well known that the intervals gradually shorten, and that the paroxysms leave more and more evident traces of their ravages behind them, till at last the joints become enlarged, stiffened, contracted, and disfigured by calcareous depositions. This catastrophe, however, is pretty frequently anticipated by the occurrence of apoplexy, paralysis, epilepsy, or organic disease of some of the more important viscera—by stone, jaundice, dropsy, &c. Thus the sufferer has not only his own peculiar malady to contend with, but the morbid predisposition to other diseases engendered by this gouty diathesis, and which, if not corrected by suitable regimen, is likely to conduct him to some fatal malady. The complicated histories of gout, with which medical writings abound, are, in Dr. Barlow's opinion, (and we are disposed to agree with him,) "only the endless modifications of the foregoing more simple series of morbid phenomena, to which the differences of age, temperament, modes of life, and other influential accompaniments give rise."

"Viewed either abstractedly or in the aggregate, they exhibit essentially only a plethoric state of constitution passing into one of febrile excitement, and ending in specific partial inflammation. The local consequences of such inflammation are but of secondary importance, for if by judicious medical discipline the constitutional plethora be corrected, so as to avert or mitigate the ensuing inflammation, and if this, when it occurs, receive the decided relief which medical treatment is capable of affording, these consequences will either be wholly prevented, or rendered so trifling as to cause little injury. When it is considered that gout may arise under every state of plethora described in the early part of this essay, and that its natural progress has been disturbed, and rendered intricate by greater diversity of treatment than most other diseases have experienced, there will be no difficulty in comprehending how its pathology has been rendered complex and obscure, and its treatment unsteady, contradictory, and unsuccessful." 71.

When gout arises, for the first time, in a constitution otherwise healthy and vigorous, and consequently under what he has described as the first condition of plethora, he has no hesitation in employing the same constitutional treatment that would be resorted to under other active inflammation. In such cases there is a full bounding pulse, hot skin, white tongue, and all the concomitants of active fever. The local inflammation, therefore, he thinks, so far from militating against active depletion, affords a strong reason for promptly resorting to it—"for if not speedily allayed by suitable depletion, severe pain is unnecessarily prolonged, disorganization is hazarded, and greater debility of the parts affected ensues."

"On the first occurrence, therefore, of a gouty paroxysm in an otherwise healthy subject, a full blood-letting should be employed, and the bowels should be freely purged with a competent dose of calomel and antimony, followed by a saline cathartic. If these means succeed in making impression on the system, so as to reduce the pulse nearly to the healthy standard, the general fever should be treated by salines, antimonials, and aperients, administered as in any other febrile excitement. If the pulse again rise in frequency, strength, or hardness, with increased heat of skin, and renewed whiteness of tongue, a repetition of blood-letting and purging with be required." 75.

The only distinction Dr. Barlow is inclined to make between such an attack and any of the simple phlegmasiæ, would be in the topical treatment—"for I would not abstract blood locally under such circumstances, nor by any direct repellants interfere with the process instituted by Nature, further than the urgency of suffering might demand, which will seldom be extreme, if the constitutional treatment be carried to the necessary extent."

"The converse of this practice, or that which, disregarding the constitutional derangement, directs its efforts chiefly to subduing the local inflammation, has been recommended at different times, and revived of late years, but no practice can be more hazardous, or more at variance with sound pathology." 77.

Were incipient gout, observes our author, always treated by vigorous constitutional means, we should have few victims of reiterated gout, and the disease would soon cease to be the opprobrium medicorum.

When, however, gout arises in habits of inferior vigour, vitiated by other diseases, or broken by the repeated accessions of gout itself, the treatment, though similar in principle, must be greatly modified.

"Before dismissing this part of the subject, I wish to discuss the merits, and point out the proper application, of colchicum, one of the most powerful auxiliaries in the treatment of gout with which I am acquainted. A full dose of this medicine purges copiously, allays pain, and lowers the pulse. These effects are produced with greater certainty if the fulness of circulation be previously reduced by blood-letting, and the mucous secretions of the intestines evacuated. When inflammation is high, as marked by a strong bounding pulse, hot skin, and loaded tongue, blood-letting should always precede the use of colchicum. But in cases where arterial action is more moderate, and direct depletion from any cause questionable, this medicine may be resorted to with peculiar propriety, and eminent advantage. Its operation seems to combine the several advantages of bleeding, purging, and sedatives; and is, therefore, particularly adapted to those cases where active depletion is inexpedient." 80.

Of the various preparations of colchicum, Dr. Barlow prefers the vinous and spirituous tinctures of the seeds, as being more uniform in strength, and more certain in operation. In cases of active gout occurring in full habits, our author invariably bleeds, and purges with calomel and antimony, before he has recourse to colchicum.

"When colchicum is to be employed, it may be given either in full doses, so as to purge actively, or in divided doses frequently repeated. A dram, dram and a half, or two drams of the tincture of the seeds should be administered at night, and repeated, if necessary, next morning. This quantity will generally purge briskly; but if it fail, a third dose the following night will pretty generally succeed; at least I have seldom found it necessary to exceed these quantities. The full operation being thus obtained, I usually continue its use in smaller doses, ordering twenty drops or minims three times a-day in any of the common saline mixtures. Even this dose will occasionally purge so actively as in a short time to require its discontinuance; in which case, the antimonial salines should be given without it, as long as febrile symptoms render necessary." 84.

By such means as the above, Dr. Barlow observes, a paroxysm of gout is effectually relieved, the constitution re-established, the powers of the limb preserved, and the gouty disposition diminished, forming, he thinks, a striking contrast with the inert treatment so long in fashion, by which the paroxysm is prolonged, the constitution imperfectly relieved, the structure of the joints injured, and the gouty diathesis confirmed. But, as was before hinted, this degree of active treatment is only applicable to simple forms of the disease, in vigorous constitutions. The same principles and treatment must be modified to the various inferior grades of the disease by the judgment of the practitioner.

Our author next proceeds to the medical treatment of the third condition of plethora, the general phenomena of which are, a sallow aspect and dusky skin; low pulse, soft and compressible; surface of the body harsh, dry, and unperspirable; tongue moist, clean, red; appetite capricious, often craving and voracious, with an endless train of dyspeptic symptoms; alvine discharges foul, dark, slimy, pitch-like, and not exhibiting the appearance of real fæces; urine high-coloured, depositing more or less of dark fetid sediment; decline of flesh and strength. "The condition itself I believe to arise from the imperfect discharge of excrementitious matters; and the depraved state of the several excretions I regard as resulting from the laboured, though ineffectual efforts of the constitution to accomplish its own purification."

Our author is aware that several of the symptoms above enumerated would be considered indicative of disordered function of the liver—and he admits that this organ is disordered in function, in common with the whole alimentary canal. But he denies that the symptoms point to organic affection, or are owing to any *primary defect*, but are owing to the effort of the constitution to purify a vitiated mass of infirmities. We confess that we do not clearly understand this pathology, and that we are far more inclined to take the simple view of the subject which attributes a good deal of the constitutional disturbance to this functional disorder of the chylopoietic viscera.

"Accordingly as more or less of nutritive plethora becomes combined with this condition, the constitutional efforts increase, and various degrees of febrile and inflammatory excitement ensue. In proportion as this excitement is energetic, and as measures of suitable activity are employed for its relief, so is the vitiated state of the blood corrected, the secretions and excretions improved, while general health and strength amend. The increased secretions from the bowels seem to be the natural discharge by which nature aims at getting rid of such impurities. To promote them, therefore, by suitable purgatives, at the same time supporting strength with a lightly nutritive diet, is the first indication." 91.

When relief, to a certain extent, is thus afforded, the powers of the constitution rally, and a febrile effort is made to assist in expediting the work of purification. As this advances, depletion should be more active, and the diet less stimulating. When sufficient excitement exists to warrant the employment of blood-letting, we may then consider the curative process in the most favourable train. During the early languor, however, of this condition, various tonics and stimulants are found highly beneficial; and our author thinks there is no auxiliary remedy more salutary or better suited to afford relief than the Bath Waters.

"By their external use they purify the skin, and thus tend to restore one most important excretory to a state of greater efficiency; while by their tonic and stimulant properties, so grateful to the stomach, and invigorating to the whole frame, they support it under those curative efforts which medical skill ought to foster and promote." 93.

Dr. Barlow thinks that the more general introduction of vapour baths into this country, such as Mr. Wallace has erected in Dublin, will be of great benefit in the treatment of this class of maladies, as well as of many others. But the great superiority of the Bath Waters consists, he observes, in the peculiar tonic and stimulating properties which they possess when internally administered. Independent of their effects on the stomach and bowels, they promote diuresis, and thus call into increased activity another excretory for depurating the mass of circulating fluids; while by exciting the whole vascular system they powerfully assist the efforts of Nature, in forming the stage of mild febrile excitement, which, as has been formerly observed, forwards more rapidly the work of purification.

Our author returns again to the subject of gout, but we cannot rest any longer on this topic. We, shall, however, introduce the following extract before we pass on to other matters.

"Ere the subject of gout is finally dismissed, I wish to offer a few remarks on the effects of mercury in this disease. It is a well known fact that mercury, taken to the extent of affecting the system, will, in gouty habits, bring on a paroxysm; and the fit thus excited is generally severe. This circumstance seems to have created a strong distrust of mercury, as a remedy in this complaint; and one of our latest writers expresses himself in strong terms respecting the hazard attending its use. My own experience confirms the facts, both of mercury exciting gout, and of the ensuing paroxysm being unusually violent: but it has led me to draw from them a very different conclusion; for I am confident that if such paroxysms are treated on the principles laid down in this work, the recovery, far from being protracted, will be as speedy as under any other circumstances; and that subsequently the constitution will be much more relieved than by a spontaneous paroxysm, though if such attack be consigned to the ordinary course of negative treatment, considerable ravages, and a tedious recovery may undoubtedly be expected,

"The fact appears to me valuable from evincing a power in mercury of exciting gouty action, such as may, on suitable occasion, be beneficially employed. Its indiscriminate use would, of course, be as unwise as abstaining wholly from its employment, merely because under improper management injury had arisen." 106.

Eight cases of gout illustrative of our author's pathological doctrines are given, and to the work itself we must refer for their details.

*Rheumatism.* In its highest and most acute form, our author observes, there are few diseases which require more decisive bleeding and other antiphlogistic measures. We cannot agree with him, however, that we have the same control over acute rheumatism that we have over the other phlegmasiæ.

"The early depletion should be carried to the extent of making due impression on the constitution, with little regard to quantity. By this means only can blood be ultimately saved; for a single evacuation early in the disease, carried to thirty or forty ounces, and producing syncope, will act more powerfully in subduing inflammation, than one dozen bleedings drained away in small and inadequate quantities. If, at the onset of disease, impression be made by the depletion here recommended, followed up by suitable purging, conducted on the principles formerly laid down, and by other auxiliary remedies, the disease will, in most cases, be speedily brought under perfect control, and one or two small additional bleedings will complete the cure. On the contrary, by trusting to trifling blood-lettings, in the beginning, the inflammation is unrestrained, disorganization goes forward, the general powers sink, and the patient is consigned for life to irremediable decrepitude, if not cut off by a metastasis of inflammation to some vital part." 118.

Our author properly states, that "such active practice is only applicable to those extreme cases which will not yield to milder measures." "In by far the larger proportion of cases much milder means will suffice." The following methodus medendi we can recommend as far preferable, in general, to that by enormous venesections.

"In ordinary cases, I consider from sixteen to twenty ounces a full blood-letting; and in many which occur in the different degrees of the second condition of plethora, twelve ounces will frequently suffice: when carried to the extent of inducing syncope, occasioning perspiration to break forth, or causing the pulse sensibly to falter, a sufficient effect may be considered produced. Soon afterwards a full dose of calomel and antimony should be given, and in due time a purging draught. If the violence of attack or intensity of suffering demand it, the calomel should be administered immediately after the venesection, and a purging draught in three or four hours; but if there be room for slight delay, I consider it peculiarly advantageous for several reasons, readily recognised by the experienced practitioner, to give the calomel at night and the draught next morning.

"The term full dose, when applied to calomel, is, perhaps, too indefinite; the practice of warm climates having of late years rendered scruple or half-dram doses quite familiar to the ear. Such quantities, however, are very rarely required in this country, and I seldom find occasion to prescribe more than five grains at once, though much larger quantities may, no doubt, be given with perfect safety. This quantity, conjoined with three or five grains of antimonial powder, or a grain of emetic tartar, is abundantly active for all ordinary purposes, and when followed by a suitable cathartic draught, will seldom fail to purge both stomach and bowels effectually.

"The latter being thus thoroughly cleared, colchicum may be freely employed when purging is again required, and in the mean time it should be exhibited, in smaller quantities, conjoined with any ordinary saline febrifuge. I have elsewhere remarked, that I consider from one dram to two of the wine or tincture of the seeds a full dose, and twenty minims a divided one. It acts in smaller quantity, and with more effect, when the bowels have been previously unloaded of their mucous secretions by calomel and antimony." 121.

It too often happens, as Dr. Barlow justly observes, that the disease is not so easily arrested—that inflammatory fever continues—that the local inflammation shifts or extends—or even metastases to vital organs occur.

"The proper means of treating this disease, under such circumstances, demands serious attention; for by confining the treatment to simple antiphlogistic regimen, however active, disappointment often arises, and recovery becomes tedious. How far the powers of colchicum, which are considerable in assuaging pain, and lowering the pulse, will supply the deficiency of bleeding and purging, I have not experience enough of its efficacy in this respect absolutely to pronounce; but, from what I have witnessed, I believe it capable of accomplishing much. Prior to colchicum acquiring its present well-deserved reputation, my chief reliance in such obstinate rheumatism as I have just described, was on calomel and opium, employed in the manner prescribed about sixty years ago by Dr. Robt. Hamilton, of Lynn-Regis, and lately noticed with so much approbation by Dr. John Armstrong, in his truly valuable work on typhus fever. By means of calomel, conjoined with opium, given in divided doses, and continued at suitable intervals until the gums become tender, the progress of rheumatic inflammation is arrested, and the disease brought to a termination relatively speedy. My experience of this treatment leads me to recommend it with much confidence, not only in rheumatism, but in any other inflammation, local or general, which resists the ordinary treatment. In acute rheumatism, I would commence with bleeding, purging, and the general antiphlogistic regimen; and if under the active use of these remedies, conjoined with colchicum, impression were not speedily made on the disease, so as to arrest the successive transitions of inflammation, I would not hesitate to bring the constitution under the influence of mercury, by means of calomel and opium. The advantage of conjoining the latter with calomel is two-fold, it acting as a sedative in allaying pain, and by its astringency restraining the purgative effects of calomel, of which the more certain and speedy absorption is thus ensured." 123.

We can corroborate the above statement by a very considerable range of experience in this distressing disease; and to a neglect of this mode of treatment, we attribute many of those fatal translations of rheumatic inflammation to the heart, which have attracted attention of late years.

The obstinacy of chronic rheumatism, and the celebrity which the Bath Hospital has attained in its treatment, induce us to extract the following account of the methodus medendi pursued in the establishment abovementioned.

"When patients are admitted under chronic rheumatism, if no prominent distress present itself, nor any high degree of inflammation prevail, the bowels are opened, and the bath is prescribed. Should whiteness of tongue and a quick pulse exist, a solution of Epsom salt and tartar emetic is given as an ordinary saline. This gentle aperient abates fever, lowers the pulse, cleanses the tongue, and enables the patient to bear the stimulant effects of the Waters. Under this treatment, essential relief is often obtained. Should local suffering, however, not be abated by these means, if the pulse rise, the skin become hot, and the tongue whiter and more furred, some blood is taken, a dose of calomel and a purging draught are prescribed, followed by a saline mixture with colchicum. The quantity of blood taken is influenced by the patient's strength and the degree of inflammatory action, and is generally from four to twelve ounces. Should the constitutional disease still resist, mercury is resorted to, and either a blue pill is given every night, a Plummer's pill night and morning; or, if more active measures be required, the patient is confined to bed, and small doses of calomel and antimony are administered at intervals until the gums become tender. The mercurial effect is kept up for a short time, after which free purging is employed, the bathing renewed, and either bark conjoined with nitre, or decoction of bark with colchicum, completes the cure." 130.

For the stiffness and enlargement of joints that often remain after pain is removed, they found nothing more serviceable than repeated blistering, frictions with tartar emetic ointment or unguentum hydrargyri, champooing, &c.

*Consideration of Palsy.* Palsy is a disease, our author remarks, which is signally benefitted by the Bath Waters. It is now generally granted that the cause of paralysis, as of apoplexy, is the effusion of blood from some ruptured vessel, or deposition within the substance of the brain or spinal marrow. As perfect recovery can rarely be looked for in serious cases, we can only endeavour, as much as possible to improve the muscular power, " in which respect the Bath Waters are proved by ample experience to accomplish more than any other remedy." The following extract will shew the mode of treatment pursued in the Bath Hospital.

" When paralytic patients are admitted into the Bath Hospital, my first care is to provide against a renewal of attack, by reducing plethora, and moderating febrile excitement, if manifested by their appropriate indications. A quick and hard pulse, hot skin, and white tongue, call invariably for active depletion. Blood is therefore taken, the bowels are purged, and salines, antimonials, and colchicum administered. When the congestive or inflammatory state is thus reduced, then, but not till then, are the Waters permitted to be used. If febrile excitement is found to continue after such treatment, adequately pursued, small doses of mercury are employed, to assist the constitutional efforts, while blood-letting and purging are renewed as increase of vascular action requires.

" The derangements of the constitution being corrected by such means, the paralytic debility next engages its necessary portion of attention; and so powerful are the Bath Waters in relieving it, that to doubt their efficacy would be blindly and wilfully to disregard the host of evidence which the practice of the Bath Hospital daily supplies." 163.

Eruptive diseases are among those in which the utility of the Bath Waters becomes conspicuous. While our author gives every credit to those distinguished dermologists (Willan, Alibert, Plenck, Bateman, &c.) who have displayed so much ingenuity in discriminating the several species of eruptions, still, he thinks, it must be confessed " that the distinctions so minutely drawn by these distinguished nosologists have alone but little to improve our practice in these diseases; and as far as regards the great proportion of chronic eruptions, the simple distinction of indolent and irritable, seems to be that by which the practical treatment is most beneficially influenced." So long, he observes, as the general constitution is deranged, it will be in vain, or even unsafe to attempt the cure of chronic eruptions. The first object therefore, is to improve the general constitution, when the local disease will readily give way—except in some chronic eruptions of an inveterate nature.

"Conformably with these views, whenever eruptive cases come under my care, I invariably proceed to amend the general health ere I resort to any specific local treatment, the Bath Waters alone excepted. If a quick pulse and white tongue denote febrile excitement, I direct bleeding, purging, and febrifuge remedies, in conjunction with the bath. When by such means plethora is removed and febrile action abated, I then avail myself of such local treatment as is best suited to each specific malady. In these diseases, mercury, administered either alone or combined with antimony, is peculiarly serviceable. Warm bathing is of considerable efficacy; and I have little doubt that still higher advantages would result if we possessed a course of bathing discipline corresponding to the Russian vapour-bath, and capable of dislodging from the surface the load of dry and hardened cuticle by which transpiration is so greatly impeded. A valuable addition seems to be made to our remedies for cutaneous complaints, in the vapour-bath of Mr. Wallace, of Dublin, which I formerly noticed." 167.

Dr. Barlow's work concludes with an appendix containing a recapitulation of his physiological and pathological principles, condensed in a series of distinct propositions. But this part we need not touch upon, after the very full, and we hope satisfactory analysis which we have given of the whole work.

